# 2-Benzyl­imino­meth­yl-6-bromo-4-chloro­phenol

**DOI:** 10.1107/S1600536808024884

**Published:** 2008-08-09

**Authors:** Xiao-Hua Pu

**Affiliations:** a Department of Chemistry, Baoji University of Arts and Science, Baoji, Shaanxi 721007, People’s Republic of China

## Abstract

The title mol­ecule, C_14_H_11_BrClNO, adopts a *trans* configuration with respect to the C=N double bond. The dihedral angle between the two aromatic rings is 70.4 (5)°. An intra­molecular O—H⋯N hydrogen bond is observed between the hydroxyl and imine groups.

## Related literature

For related literature, see: Ali *et al.* (2002 ▶); Cukurovali *et al.* (2002 ▶); Tarafder *et al.* (2002 ▶). For bond-length data, see: Allen *et al.* (1987 ▶).
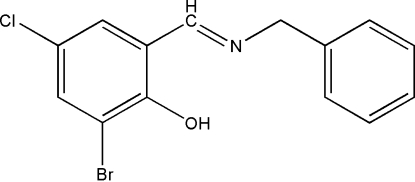


## Experimental

### 

#### Crystal data

C_14_H_11_BrClNO
*M* *_r_* = 324.60
Monoclinic, 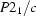

*a* = 4.3334 (8) Å
*b* = 12.8976 (14) Å
*c* = 23.892 (2) Å
β = 92.992 (1)°
*V* = 1333.5 (3) Å^3^
*Z* = 4
Mo *K*α radiation
μ = 3.27 mm^−1^
*T* = 298 (2) K
0.40 × 0.37 × 0.13 mm

#### Data collection

Bruker SMART CCD area-detector diffractometer
Absorption correction: multi-scan (*SADABS*; Sheldrick, 1996 ▶) *T*_min_ = 0.355, *T*_max_ = 0.676
6753 measured reflections
2325 independent reflections
1699 reflections with *I* > 2σ(*I*)
*R*_int_ = 0.068

#### Refinement

*R*[*F* ^2^ > 2σ(*F* ^2^)] = 0.067
*wR*(*F* ^2^) = 0.176
*S* = 1.06
2325 reflections
163 parameters
H-atom parameters constrained
Δρ_max_ = 1.47 e Å^−3^
Δρ_min_ = −0.76 e Å^−3^

### 

Data collection: *SMART* (Bruker, 2000 ▶); cell refinement: *SAINT* (Bruker, 2000 ▶); data reduction: *SAINT*; program(s) used to solve structure: *SHELXS97* (Sheldrick, 2008 ▶); program(s) used to refine structure: *SHELXL97* (Sheldrick, 2008 ▶); molecular graphics: *SHELXTL* (Sheldrick, 2008 ▶); software used to prepare material for publication: *SHELXTL*.

## Supplementary Material

Crystal structure: contains datablocks I, global. DOI: 10.1107/S1600536808024884/ci2649sup1.cif

Structure factors: contains datablocks I. DOI: 10.1107/S1600536808024884/ci2649Isup2.hkl

Additional supplementary materials:  crystallographic information; 3D view; checkCIF report

**Fig. 1.**
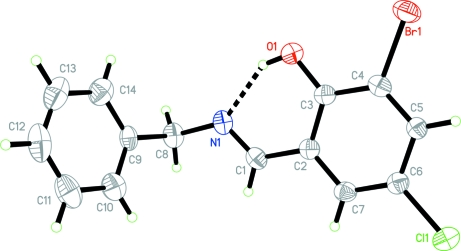
The molecular structure of the title compound, showing 30% probability ellipsoids. The dashed line represents a hydrogen bond.

Enhanced figure: interactive version of Fig. 1

## Figures and Tables

**Table 1:** Hydrogen-bond geometry (Å, °)

*D*—H⋯*A*	*D*—H	H⋯*A*	*D*⋯*A*	*D*—H⋯*A*
O1—H1⋯N1	0.82	1.86	2.590 (7)	147

**Fig. 2.**
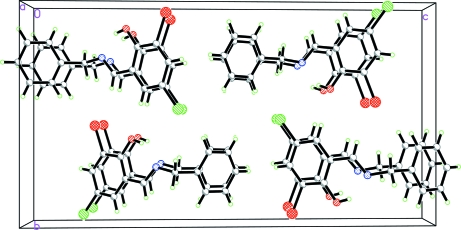
The crystal packing of the title compound, viewed down the b axis.

## supplementary crystallographic information

### Comment

Schiff base compounds have been of great interest for many years. These compounds play an important role in the development of coordination chemistry related to catalysis and enzymatic reactions, magnetism and molecular architectures. As an extension of the work on the structural characterization of Schiff base compounds, the crystal structure of the title compound is reported here.

Bond lengths in the title molecule (Fig. 1) have normal values (Allen *et al.*,1987). The C1═N1 bond length of 1.267 (9) Å conforms to the value for a double bond. The dihedral angle between the two aromatic rings is 70.4 (5)°. As expected, the molecule adopts a trans configuration about the C═N bond [C8—N1—C1—C2 = -178.6 (6)°]. An intramolecular O—H···N hydrogen bond is observed between hydroxyl and imine groups (Table 1).

### Experimental

3-Bromine-5-chlorosalicylaldehyde (0.1 mmol, 23.55 mg) and 1-benzylamine (0.1 mmol, 10.7 mg) were added to methanol (10 ml). The mixture was stirred for 30 min at room temperature to give a clear brown solution. After allowing the resulting solution to stand in air for 7 d, yellow block-shaped crystals of the title compound were formed on slow evaporation of the solvent. The crystals were collected, washed with methanol and dried in a vacuum desiccator using anhydrous CaCl_2_ (yield 54%). Analysis found: C 51.76, H 4.0%; calculated for C_14_H_11_Br_Cl_N_O_: C 51.77, H 3.39%.

### Refinement

All H atoms were placed in geometrically idealized positions [O-H = 0.82 Å and C-H = 0.93–0.97 Å] and constrained to ride on their parent atoms, with *U*_iso_(H) = 1.2*U*_eq_(C) and 1.5*U*_eq_(O). The highest unassigned peak in the difference map is located 0.85 and 1.05 Å  from atoms Cl1 and C6, respectively.

### Crystal data

**Table:**

C_14_H_11_BrClNO	*F*_000_ = 648
*M**_r_* = 324.60	*D*_x_ = 1.617 Mg m^−^^3^
Monoclinic, *P*2_1_/*c*	Mo *K*α radiation λ = 0.71073 Å
Hall symbol: -P 2ybc	Cell parameters from 2314 reflections
*a* = 4.3334 (8) Å	θ = 3.0–23.6º
*b* = 12.8976 (14) Å	µ = 3.27 mm^−^^1^
*c* = 23.892 (2) Å	*T* = 298 (2) K
β = 92.992 (1)º	Block, yellow
*V* = 1333.5 (3) Å^3^	0.40 × 0.37 × 0.13 mm
*Z* = 4	

### Data collection

**Table:**

Bruker SMART CCD area-detector diffractometer	2325 independent reflections
Radiation source: fine-focus sealed tube	1699 reflections with *I* > 2σ(*I*)
Monochromator: graphite	*R*_int_ = 0.068
*T* = 298(2) K	θ_max_ = 25.0º
φ and ω scans	θ_min_ = 1.7º
Absorption correction: multi-scan(SADABS; Sheldrick, 1996)	*h* = −5→5
*T*_min_ = 0.355, *T*_max_ = 0.676	*k* = −14→15
6753 measured reflections	*l* = −28→19

### Refinement

**Table:**

Refinement on *F*^2^	Secondary atom site location: difference Fourier map
Least-squares matrix: full	Hydrogen site location: inferred from neighbouring sites
*R*[*F*^2^ > 2σ(*F*^2^)] = 0.067	H-atom parameters constrained
*wR*(*F*^2^) = 0.176	*w* = 1/[σ^2^(*F*_o_^2^) + (0.0638*P*)^2^ + 4.7838*P*] where *P* = (*F*_o_^2^ + 2*F*_c_^2^)/3
*S* = 1.06	(Δ/σ)_max_ = 0.001
2325 reflections	Δρ_max_ = 1.47 e Å^−^^3^
163 parameters	Δρ_min_ = −0.76 e Å^−^^3^
Primary atom site location: structure-invariant direct methods	Extinction correction: none

### Special details

**Table:**

Geometry. All e.s.d.'s (except the e.s.d. in the dihedral angle between two l.s. planes) are estimated using the full covariance matrix. The cell e.s.d.'s are taken into account individually in the estimation of e.s.d.'s in distances, angles and torsion angles; correlations between e.s.d.'s in cell parameters are only used when they are defined by crystal symmetry. An approximate (isotropic) treatment of cell e.s.d.'s is used for estimating e.s.d.'s involving l.s. planes.
Refinement. Refinement of *F*^2^ against ALL reflections. The weighted *R*-factor *wR* and goodness of fit *S* are based on *F*^2^, conventional *R*-factors *R* are based on *F*, with *F* set to zero for negative *F*^2^. The threshold expression of *F*^2^ > σ(*F*^2^) is used only for calculating *R*-factors(gt) *etc*. and is not relevant to the choice of reflections for refinement. *R*-factors based on *F*^2^ are statistically about twice as large as those based on *F*, and *R*- factors based on ALL data will be even larger.

### Fractional atomic coordinates and isotropic or equivalent isotropic displacement parameters (Å^2^)

**Table:**

	*x*	*y*	*z*	*U*_iso_*/*U*_eq_	
Br1	1.1330 (2)	0.05445 (6)	0.34840 (4)	0.0688 (3)	
Cl1	1.1495 (6)	0.47661 (15)	0.37758 (10)	0.0761 (6)	
N1	0.4056 (13)	0.2399 (4)	0.1816 (2)	0.0495 (14)	
O1	0.7218 (12)	0.1150 (3)	0.2472 (2)	0.0588 (13)	
H1	0.6061	0.1323	0.2205	0.088*	
C1	0.4902 (15)	0.3140 (6)	0.2137 (3)	0.0465 (16)	
H1A	0.4104	0.3798	0.2062	0.056*	
C2	0.7087 (14)	0.3002 (5)	0.2620 (3)	0.0409 (14)	
C3	0.8091 (15)	0.1982 (5)	0.2763 (3)	0.0419 (15)	
C4	1.0094 (15)	0.1881 (5)	0.3238 (3)	0.0442 (15)	
C5	1.1108 (15)	0.2704 (5)	0.3547 (3)	0.0425 (15)	
H5	1.2421	0.2609	0.3864	0.051*	
C6	1.0135 (16)	0.3711 (5)	0.3381 (3)	0.0476 (16)	
C7	0.8123 (16)	0.3849 (5)	0.2934 (3)	0.0481 (16)	
H7	0.7434	0.4511	0.2839	0.058*	
C8	0.1902 (16)	0.2605 (6)	0.1331 (3)	0.0562 (19)	
H8A	0.0875	0.3264	0.1380	0.067*	
H8B	0.0339	0.2066	0.1302	0.067*	
C9	0.3671 (15)	0.2630 (6)	0.0799 (3)	0.0480 (17)	
C10	0.4684 (18)	0.3549 (7)	0.0600 (3)	0.066 (2)	
H10	0.4266	0.4163	0.0786	0.080*	
C11	0.635 (2)	0.3571 (9)	0.0117 (4)	0.082 (3)	
H11	0.7064	0.4196	−0.0021	0.099*	
C12	0.691 (2)	0.2664 (10)	−0.0149 (4)	0.086 (3)	
H12	0.8006	0.2675	−0.0473	0.103*	
C13	0.594 (2)	0.1762 (9)	0.0043 (4)	0.084 (3)	
H13	0.6386	0.1154	−0.0146	0.101*	
C14	0.4270 (18)	0.1718 (7)	0.0517 (4)	0.069 (2)	
H14	0.3557	0.1087	0.0646	0.083*	

### Atomic displacement parameters (Å^2^)

**Table:**

	*U*^11^	*U*^22^	*U*^33^	*U*^12^	*U*^13^	*U*^23^
Br1	0.0795 (6)	0.0510 (5)	0.0758 (6)	0.0131 (4)	0.0027 (4)	0.0117 (4)
Cl1	0.0979 (16)	0.0525 (11)	0.0767 (14)	−0.0047 (10)	−0.0060 (12)	−0.0098 (10)
N1	0.039 (3)	0.066 (4)	0.044 (3)	0.000 (3)	0.003 (3)	0.003 (3)
O1	0.076 (3)	0.047 (3)	0.053 (3)	0.000 (2)	0.001 (2)	−0.002 (2)
C1	0.039 (4)	0.056 (4)	0.045 (4)	0.002 (3)	0.013 (3)	0.009 (3)
C2	0.039 (3)	0.046 (3)	0.040 (4)	−0.001 (3)	0.016 (3)	0.001 (3)
C3	0.042 (4)	0.042 (3)	0.043 (4)	−0.001 (3)	0.016 (3)	0.001 (3)
C4	0.040 (4)	0.049 (4)	0.045 (4)	0.004 (3)	0.019 (3)	0.008 (3)
C5	0.042 (4)	0.047 (3)	0.039 (4)	0.002 (3)	0.008 (3)	0.001 (3)
C6	0.049 (4)	0.048 (4)	0.046 (4)	−0.001 (3)	0.009 (3)	−0.003 (3)
C7	0.053 (4)	0.042 (3)	0.050 (4)	0.005 (3)	0.011 (3)	0.000 (3)
C8	0.035 (4)	0.078 (5)	0.055 (5)	0.000 (3)	−0.002 (3)	0.001 (4)
C9	0.037 (4)	0.068 (4)	0.038 (4)	0.002 (3)	−0.007 (3)	0.002 (3)
C10	0.052 (5)	0.082 (6)	0.065 (5)	0.000 (4)	−0.002 (4)	0.007 (4)
C11	0.059 (5)	0.115 (8)	0.072 (6)	0.000 (5)	−0.003 (5)	0.028 (6)
C12	0.062 (6)	0.144 (10)	0.051 (6)	0.013 (6)	0.005 (4)	0.006 (6)
C13	0.068 (6)	0.113 (8)	0.070 (6)	0.022 (6)	−0.007 (5)	−0.028 (6)
C14	0.055 (5)	0.081 (6)	0.071 (6)	0.004 (4)	−0.010 (4)	−0.010 (5)

### Geometric parameters (Å, °)

**Table:**

Br1—C4	1.890 (6)	C7—H7	0.93
Cl1—C6	1.741 (7)	C8—C9	1.518 (10)
N1—C1	1.267 (9)	C8—H8A	0.97
N1—C8	1.473 (9)	C8—H8B	0.97
O1—C3	1.325 (8)	C9—C10	1.359 (10)
O1—H1	0.82	C9—C14	1.386 (11)
C1—C2	1.465 (9)	C10—C11	1.392 (12)
C1—H1A	0.93	C10—H10	0.93
C2—C7	1.387 (9)	C11—C12	1.360 (14)
C2—C3	1.422 (9)	C11—H11	0.93
C3—C4	1.397 (9)	C12—C13	1.326 (14)
C4—C5	1.353 (9)	C12—H12	0.93
C5—C6	1.416 (9)	C13—C14	1.377 (13)
C5—H5	0.93	C13—H13	0.93
C6—C7	1.355 (9)	C14—H14	0.93
			
C1—N1—C8	119.5 (6)	N1—C8—H8A	109.7
C3—O1—H1	109.5	C9—C8—H8A	109.7
N1—C1—C2	122.7 (6)	N1—C8—H8B	109.7
N1—C1—H1A	118.7	C9—C8—H8B	109.7
C2—C1—H1A	118.7	H8A—C8—H8B	108.2
C7—C2—C3	120.7 (6)	C10—C9—C14	119.8 (8)
C7—C2—C1	120.6 (6)	C10—C9—C8	119.9 (7)
C3—C2—C1	118.7 (6)	C14—C9—C8	120.3 (7)
O1—C3—C4	120.0 (6)	C9—C10—C11	119.9 (9)
O1—C3—C2	123.2 (6)	C9—C10—H10	120.0
C4—C3—C2	116.9 (6)	C11—C10—H10	120.0
C5—C4—C3	122.7 (6)	C12—C11—C10	118.9 (10)
C5—C4—Br1	117.8 (5)	C12—C11—H11	120.6
C3—C4—Br1	119.4 (5)	C10—C11—H11	120.6
C4—C5—C6	118.9 (6)	C13—C12—C11	121.7 (10)
C4—C5—H5	120.6	C13—C12—H12	119.1
C6—C5—H5	120.6	C11—C12—H12	119.1
C7—C6—C5	120.7 (6)	C12—C13—C14	120.6 (10)
C7—C6—Cl1	120.7 (5)	C12—C13—H13	119.7
C5—C6—Cl1	118.6 (5)	C14—C13—H13	119.7
C6—C7—C2	120.1 (6)	C13—C14—C9	119.0 (9)
C6—C7—H7	120.0	C13—C14—H14	120.5
C2—C7—H7	120.0	C9—C14—H14	120.5
N1—C8—C9	109.6 (5)		
			
C8—N1—C1—C2	−178.6 (6)	C5—C6—C7—C2	−2.7 (10)
N1—C1—C2—C7	175.2 (6)	Cl1—C6—C7—C2	179.2 (5)
N1—C1—C2—C3	−5.7 (9)	C3—C2—C7—C6	0.7 (10)
C7—C2—C3—O1	−178.8 (6)	C1—C2—C7—C6	179.8 (6)
C1—C2—C3—O1	2.1 (9)	C1—N1—C8—C9	102.8 (7)
C7—C2—C3—C4	1.2 (9)	N1—C8—C9—C10	−94.4 (8)
C1—C2—C3—C4	−177.9 (5)	N1—C8—C9—C14	85.3 (8)
O1—C3—C4—C5	178.9 (6)	C14—C9—C10—C11	−0.7 (11)
C2—C3—C4—C5	−1.1 (9)	C8—C9—C10—C11	179.0 (6)
O1—C3—C4—Br1	−3.9 (8)	C9—C10—C11—C12	0.4 (12)
C2—C3—C4—Br1	176.1 (4)	C10—C11—C12—C13	−0.6 (14)
C3—C4—C5—C6	−0.8 (9)	C11—C12—C13—C14	1.0 (14)
Br1—C4—C5—C6	−178.1 (5)	C12—C13—C14—C9	−1.2 (13)
C4—C5—C6—C7	2.7 (10)	C10—C9—C14—C13	1.1 (11)
C4—C5—C6—Cl1	−179.1 (5)	C8—C9—C14—C13	−178.6 (7)

### Hydrogen-bond geometry (Å, °)

**Table:**

*D*—H···*A*	*D*—H	H···*A*	*D*···*A*	*D*—H···*A*
O1—H1···N1	0.82	1.86	2.590 (7)	147
